# Prevalence of Acute Gastroenteritis Enteropathogens Among Hospitalized Children in Jordan: A Single-Center Study

**DOI:** 10.3390/v17050657

**Published:** 2025-04-30

**Authors:** Ashraf I. Khasawneh, Nisreen Himsawi, Ashraf Sammour, Faten A. Bataineh, Mohammad H. Odeh, Mayar S. Alhieh, Nawal S. Hijjawi, Mohammad Wahsheh, Hafez Al-Momani, Moureq R. Alotaibi, Sofian Al Shboul, Tareq Saleh

**Affiliations:** 1 Department of Microbiology, Pathology, and Forensic Medicine, Faculty of Medicine, The Hashemite University, Zarqa 13133, Jordan; 2 Department of Anatomy, Physiology and Biochemistry, Faculty of Medicine, The Hashemite University, Zarqa 13133, Jordan; 3 Laboratory Department, Princess Rhama Hospital, Ministry of Health, Irbid 21110, Jordan; 4 Department of Medical Laboratory Sciences, Faculty of Applied Health Sciences, Hashemite University, Zarqa 13133, Jordan; 5 Department of Pediatrics, Princess Rhama Hospital, Ministry of Health, Irbid 21110, Jordan; 6 Department of Pharmacology and Toxicology, College of Pharmacy, King Saud University, Riyadh 12372, Saudi Arabia; 7 Department of Pharmacology and Public Health, Faculty of Medicine, The Hashemite University, Zarqa 13133, Jordan; 8 Department of Pharmacology & Therapeutics, College of Medicine & Health Sciences, Arabian Gulf University, Manama P.O. Box 26671, Bahrain

**Keywords:** acute gastroenteritis, rotavirus, norovirus, adenovirus, Jordan

## Abstract

Background and objectives: Acute gastroenteritis (AGE) remains a significant cause of morbidity in children, particularly in low- and middle-income countries. Viral pathogens, including rotavirus (RoV), norovirus (NoV), and adenovirus (HAdV), are among the leading causes of AGE. This study aimed to determine the prevalence of viral, bacterial, and parasitic enteric pathogens associated with AGE among hospitalized children in Northern Jordan. Materials and Methods: A total of 195 stool samples were collected from hospitalized children with AGE during the winter seasons of 2022–2024. Multiplex real-time qPCR assays were performed to detect common pathogens. The prevalence of each pathogen was determined, and co-infections were analyzed. Clinical symptoms, demographic characteristics, and associations between specific pathogens and disease severity were evaluated. Results: Viral pathogens were the predominant cause of AGE, with NoV detected in 53 cases (27.2%; of which 19.0% were NoV GI and 8.2% NoV GII), followed by RoV (24.1%), HAdV (20.0%), HAstV (13.3%), and SaV (12.3%). Co-infections were observed in several cases, particularly among viral infections evoked by RoV, HAdV, and NoV GI. Bacterial and parasitic infections were less prevalent, with *Salmonella* and *Campylobacter* spp. detected in 23.1% and 13.8%, respectively. Additionally, *Cryptosporidium* was identified in two cases (0.5%). Conclusions: Viral pathogens, particularly NoV, RoV, and HAdV, are the leading causes of AGE among hospitalized children in Jordan. Co-infections among viral pathogens were common, whereas bacterial and parasitic infections played a limited role in the disease burden. These findings emphasize the importance of continued surveillance and vaccination efforts, particularly for RoV, to reduce AGE-related hospitalizations in children.

## 1. Introduction

Diarrheal diseases pose a significant global health burden, particularly among children under five years old. While the mortality rates are the highest in Sub-Saharan Africa and South Asia, diarrhea also imposes substantial medical costs in high-income countries [1,2,3]. Viral diarrhea affects individuals of all ages, with young children being particularly vulnerable. Rotavirus remains a leading cause of severe acute gastroenteritis (AGE) in children under five years of age worldwide, presenting a significant mortality risk, especially in developing nations [4]. According to the World Health Organization (WHO), approximately 440,000 children succumb annually to diarrheal illnesses associated with rotavirus, with the majority (80%) of fatalities occurring in low-income countries [5]. In 2019, rotavirus contributed to approximately 111 million cases of non-hospitalized diarrheal episodes and led to two million hospitalizations among children under five globally [4]. Rotavirus (RoV) is notably the most common cause of AGE among hospitalized children, alongside norovirus (NoV), sapovirus (SaV), human astrovirus (HAstV), and human adenovirus (HAdV), collectively responsible for most AGE cases worldwide [6].

NoV and SaV are genetically diverse, non-enveloped viruses with a single-stranded RNA genome, belonging to distinct genera within the *Caliciviridae* family [7]. NoVs are divided into ten genogroups (GI–GX), with GI and GII viruses responsible for most human infections [8]. Within these genogroups, there are further subdivisions into 48 genotypes (9 GI, 26 GII, 3 GIII, 2 GIV, 2 GV, 2 GVI, 1 GVII, 1 GVIII, 1 GIX, and 1 GX) [9]. Notably, GII.4 viruses have been linked to pandemics over the past two decades. However, other genotypes, like GII.17 and, more recently, GII.2, have emerged as predominant strains in regions such as China, Japan, and South Korea, temporarily surpassing GII.4 viruses in these areas [10]. HAdVs can cause a spectrum of illnesses, including respiratory infections, conjunctivitis, hemorrhagic cystitis, and gastrointestinal diseases. HAdV species F—specifically serotypes 40 and 41—contribute to 2.0–23.0% of acute diarrhea cases in young children, particularly infants, and are more prevalent among immunocompromised individuals [11]. These serotypes are transmitted primarily via the fecal–oral route and can lead to prolonged diarrhea (averaging 10 days), fever, vomiting, mild dehydration, and abdominal pain [12]. Infections caused by HAstV and SaV also contribute to hospitalizations and outbreaks of diarrheal illness. Globally, the prevalence of HAstV among children with AGE was found to be 4.2%, with HAstV-1 being the most common genotype, accounting for 59% [13]. Similarly, the prevalence of SaV worldwide among children with AGE was 3.4%, with genogroup I being the predominant type [14]. Several studies have indicated shifts in the prevalence of NoV, SaV, and HAstV following the introduction of the RoV vaccine among children under five years of age [15,16,17].

In addition to viral pathogens, bacterial and parasitic infections play a significant role in AGE. Among bacterial causes, *Campylobacter* species, including *C. jejuni*, *C. coli*, and *C. lari*, are frequent causes of bacterial gastroenteritis worldwide, often linked to contaminated poultry and unpasteurized dairy products [18]. *Clostridioides difficile* has emerged as an important cause of AGE, particularly in healthcare settings and in children who have received prior antibiotic treatment [19]. *Salmonella* species are another major cause of bacterial diarrhea, frequently associated with contaminated food and water. Similarly, *Shigella* species, known for their low infectious dose, cause bacillary dysentery, with *S. flexneri* and *S. sonnei* being the most prevalent serotypes in developing and developed countries, respectively [20]. *Escherichia coli* pathotypes, including verocytotoxin-producing *E. coli* (VTEC), particularly *E. coli* O157:H7, can cause severe gastroenteritis and hemolytic uremic syndrome (HUS) [21]. Additionally, *Yersinia enterocolitica* is a notable cause of AGE, often linked to contaminated pork products and associated with systemic infections in severe cases [20].

Parasitic infections also contribute to AGE, particularly in regions with poor sanitation. *Cryptosporidium* species are a leading cause of waterborne outbreaks, with *C. parvum* and *C. hominis* being the most clinically relevant [22]. *Giardia duodenalis*, a flagellated protozoan, is a well-documented cause of persistent diarrhea, particularly in children [23]. *Entamoeba histolytica*, the causative agent of amoebiasis, leads to invasive colitis and can result in extraintestinal complications, such as liver abscesses [24].

Efforts to mitigate diarrheal diseases focus on preventive measures, including rotavirus vaccination, which has significantly reduced the morbidity and mortality in many regions [16]. Additionally, improving access to clean water, sanitation, and hygiene practices remain crucial in reducing the global burden of AGE. However, the continuous surveillance of the major microbiological causes of AGE is always required.

This study was conducted at a tertiary care pediatric hospital in Irbid, Jordan, serving a large catchment area with an estimated population density of 13,720 individuals/km^2^. The region has variable access to improved sanitation, with national estimates indicating 86.0% access to basic drinking water services and 82.0% to basic sanitation services [25]. Rotavirus vaccination was introduced in Jordan’s national immunization program in 2015, with estimated national coverage rates of around 96.4% in recent years [26]. The objective of this study was to assess the prevalence of viral, bacterial, and parasitic pathogens associated with AGE among children in Northern Jordan during the winter seasons of 2022–2024. This study aimed to aid in understanding the microbial landscape contributing to acute gastroenteritis cases requiring hospitalization in a specific Jordanian pediatric population during the winter season.

## 2. Materials and Methods

### 2.1. Study Population

This study was conducted at Princess Rahma Hospital (PRH) in Irbid from October 2022 to February 2024 (Figure S1). A total of 195 stool samples were collected from 195 unique pediatric patients hospitalized with acute gastroenteritis at PRH during two winter seasons. All patients were admitted through the emergency department and met the WHO clinical criteria for AGE. Patients admitted to the pediatric ward at PRH exhibiting symptoms of AGE were enrolled in the study. Samples were obtained from children aged 9 weeks to 12 years old experiencing acute watery diarrhea. To prevent multiple freeze–thaw cycles, stool samples were divided into aliquots, stored at −80 °C, and tested for gastroenteritis viruses, bacteria, and parasites within one month of collection.

This study received Institutional Review Board (IRB) approval from The Hashemite University (No. 14/1/2021/2022) and PRH (No. MBA/IRB/8368) in accordance with established guidelines. Informed consent forms were signed by all guardians of participating children.

### 2.2. Nucleic Acid Extraction and Reverse Transcription

Viral nucleic acids were extracted from 140 µL of clarified stool suspension (10% *w*/*v*) using phosphate-buffered saline (pH = 7.2) (Thermo Scientific, Waltham, MA, USA). DNA and RNA were simultaneously extracted using the QIAamp MinElute Virus Spin Kit (Qiagen, Hilden, Germany), which allows for the concurrent isolation of both nucleic acids in a single step, following the manufacturer’s protocol. The eluted nucleic acid was stored in 100 µL of AVE buffer at −20 °C for subsequent procedures. The extraction efficiency was monitored using internal controls (ICs) provided with the real-time polymerase chain reaction (qPCR) kit (Siemens, Munich, Germany). Consistent Ct values across samples indicated efficient extraction, while deviations prompted re-extraction and rerunning (Supplementary Table S1). Following the manufacturer’s guidelines, RNA templates were used to generate complementary DNA (cDNA) strands with the QuantiTect Reverse Transcription Kit (Qiagen, Hilden, Germany). Subsequently, the cDNA samples were processed for the detection of gastrointestinal viruses using a qPCR assay.

### 2.3. Detection of Gastrointestinal Viruses in Stool Samples

To identify the virus species responsible for the children’s gastroenteritis, we conducted multiplex RT-PCR assays targeting 6 viruses using the FTD viral gastroenteritis panel (Siemens, Munich, Germany). Briefly, this FTD assay is a one-step qPCR kit that includes primer/probe mixtures for the simultaneous detection of 6 gastroenteritis viruses. These include HAdV, HAstV, NoV GI, NoV GII, RoV, SaV, and brome mosaic virus (BMV), which was included as an internal control (IC).

Three multiplex qPCR reactions were prepared according to the manufacturer’s instructions. Briefly, the first reaction involved a Noro PP tube that contained the primer/probe mixes for NoV GI, NoV GII, and IC (BMV). The second and third reactions included ARA PP tubes that contained the primer/probe mixes for HAstV, RoV, and HAdV and the Sapo PP tube that contained only the SaV primer/probe mix. Each PCR reaction was conducted at a final volume of 25 µL, with 10 μL of the cDNA combined with 15 μL of the master mix provided in the kit. The multiplex real-time qPCR thermal profile comprised an initial denaturation step at 94 °C for 1 min and then 40 cycles of denaturation at 94 °C for 8 s and annealing at 60 °C for 1 min. A sample was considered positive for a microorganism if a sigmoidal curve with a cycle threshold (Ct) value of <35 was observed. An internal control (IC) was included in the extraction process alongside the specimens and utilized in each PCR run, in addition to positive and negative controls provided by the manufacturers.

### 2.4. Detection of Gastrointestinal Bacteria and Parasites in Stool Samples

To identify the bacteria responsible for the children’s gastroenteritis, we conducted multiplex qPCR assays that included primer/probe mixtures for the simultaneous detection of 6 gastroenteritis bacteria using the FTD kit (Siemens, Munich, Germany). These included *Campylobacter coli*/*jejuni*/*lari*, *C. difficile*, *Salmonella* spp., *Shigella* spp., Verocytotoxin-producing *E. coli*, *Yersinia enterocolitica*, and murine cytomegalovirus (MCMV), which served as an internal control (IC).

Two multiplex qPCR reactions were prepared according to the manufacturer’s instructions. Briefly, the first was the CV PP primer/probe mix for verotoxin-producing *E. coli*, *C. coli*/*jejuni*/*lari*, and IC (MCMV), and the second was the CYSS PP primer/probe mix for *C. difficile*, *Salmonella* spp., *Shigella* spp., and *Y. enterocolitica*. Each PCR reaction was conducted in a final volume of 50 µL, with 10 μL of the extracted nucleic acid sample combined with 20 μL of the master mix provided in the kit. The multiplex real-time qPCR thermal conditions were similar to the ones mentioned above. An internal control (IC) was included in the extraction process alongside the specimens and utilized in each PCR run, in addition to positive and negative controls provided by the manufacturers.

Additionally, to detect parasitic infections, we employed the FTD Parasite Kit, which targets *Cryptosporidium* species, *Giardia lamblia*, and *Entamoeba histolytica*. An internal control was included in the extraction process for each sample, along with manufacturer-provided positive and negative controls, to ensure the accuracy and reliability of the PCR results.

### 2.5. Statistical Analysis

Statistical analysis was performed using the IBM SPSS Statistics software, version 24, with the Exact Test package (IBM, New York, NY, USA). Categorical variables were compared using chi-squared or Fisher’s exact tests, and *p*-values < 0.05 were considered statistically significant. Descriptive statistics (frequencies, percentages) were used to summarize demographic and clinical characteristics.

## 3. Results

### 3.1. Demographic Data and Clinical Presentation

A total of 195 children with acute gastroenteritis (AGE) were included in this study (Table 1). The majority of the patients were male (116; 59.5%), while females accounted for 79 cases (40.5%). In terms of the age distribution, children aged 2–4 years represented the largest group (60; 30.8%), followed by those ≤2 years (46; 23.6%). The remaining cases were distributed relatively evenly among the 4–6 years (30; 15.4%), 6–8 years (30; 15.4%), and >8 years (29; 14.8%) age groups.

All patients presented with diarrhea (195; 100%) at the time of sample collection, as expected for an AGE cohort. Other commonly reported symptoms included fever (130; 66.7%), vomiting (124; 63.6%), abdominal pain (97; 49.7%), and nausea (71; 36.4%). These findings highlight that AGE predominantly affects young children, with a larger proportion of cases occurring in males. Fever and vomiting were the most frequently observed symptoms, followed by abdominal pain and nausea.

### 3.2. Prevalence of Gastrointestinal Pathogens

**Figure 1:**
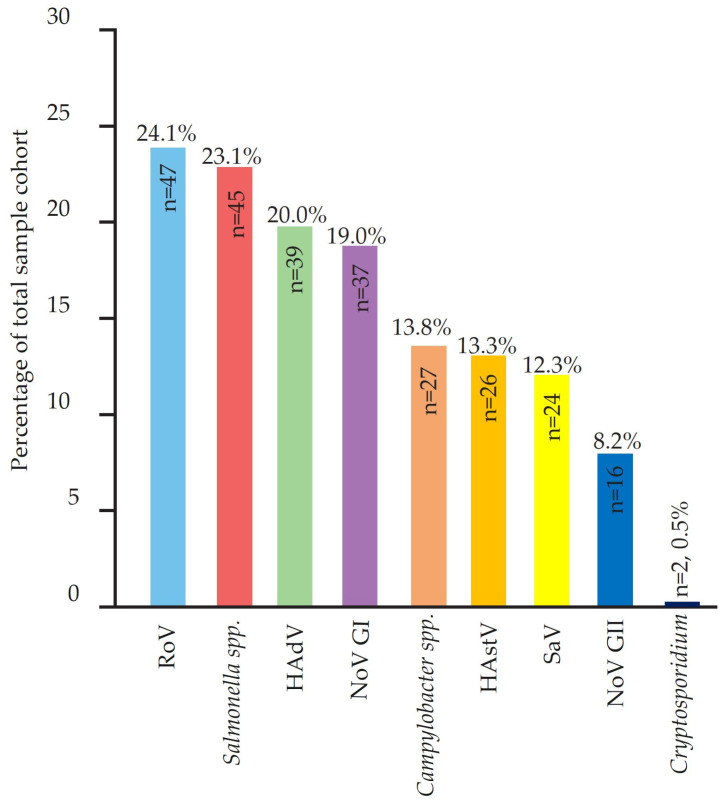
Prevalence of gastrointestinal pathogens detected among tested cases. The figure shows the number and percentage of positive cases for each pathogen. RoV: rotavirus; HAdV: human adenovirus; NoV: norovirus; HAstV: human astrovirus; SaV: sapovirus. Pathogens with no positive cases are listed for completeness. Percentages are based on the total number of samples tested.

Among the 195 children with acute gastroenteritis (AGE) included in this study, viral pathogens were the most frequently detected causative agents (Figure 1). NoV was the leading pathogen, identified in 53 cases (27.2%), followed closely by RoV (47 cases, 24.1%), *Salmonella* spp. (45 cases, 23.1%) and HAdV (39 cases, 20.0%). NoV GI was detected in 37 cases (19.0%), while NoV GII was identified in 16 cases (8.2%). HAstV (13.3%) and SaV (12.3%) were also prevalent.

Among bacterial pathogens, *Salmonella* spp. were the most frequently detected, accounting for 45 cases (23.1%). *Campylobacter* spp. were the only other bacterial pathogens detected, with a notable prevalence of 13.8%. Two samples positive for *Campylobacter* spp. had Ct values slightly above 35 (35.2 and 35.8). The melting curves for these samples were carefully analyzed and compared to those of the positive controls, confirming their validity. Notably, *C. difficile*, verocytotoxin-producing *E. coli*, *Shigella* spp., and *Yersinia enterocolitica* were not detected in any of the samples.

Parasitic infections were rare, with Cryptosporidium detected in only two cases (0.5%). Other parasites, including Entamoeba histolytica and Giardia lamblia, were not identified in any of the samples. These findings indicate that viral pathogens, particularly NoV, RoV, and HAdV, are the predominant causes of AGE in children, while bacterial infections, mainly caused by *Salmonella* spp. and *Campylobacter* spp., play a secondary role. The low prevalence of parasitic infections suggests that protozoal pathogens may not be major contributors to AGE in this population.

### 3.3. Viral and Bacterial Co-Infection Pattern

Co-infections were observed among several pathogens, with notable associations between viral pathogens (Table 2). Among the 47 RoV cases, co-infections were most frequently detected with HAdV (13 cases), NoV GI (12 cases), and HAstV (10 cases). A smaller number of RoV cases were also co-infected with SaV (three cases) and NoV GII (two cases). No co-infections of RoV with *Campylobacter* spp., *Salmonella* spp., or *Cryptosporidium* were observed.

Bacterial co-infections were less common, with *Salmonella* spp. frequently co-occurring with *Campylobacter* spp. (20 cases). Additionally, *Cryptosporidium* was detected in two cases of *Salmonella* co-infection. No other significant bacterial–bacterial or bacterial–viral co-infections were identified. Among viral co-infections, NoV GI exhibited a high frequency of co-occurrence, with 17 cases also testing positive for HAstV and nine cases for SaV. HAdV and NoV GII were found together in five cases, while HAstV and SaV co-occurred in seven cases.

Notably, *Cryptosporidium* infections were detected in only two cases, both of which were co-infected with *Salmonella* spp. These findings highlight the frequent co-infection of viral pathogens in children with AGE, particularly between RoV, NoV, HAstV, and HAdV. Bacterial co-infections were predominantly limited to *Salmonella* spp. and *Campylobacter* spp., with limited overlap with viral pathogens.

### 3.4. Age-Specific Pathogen Distribution

**Figure 2:**
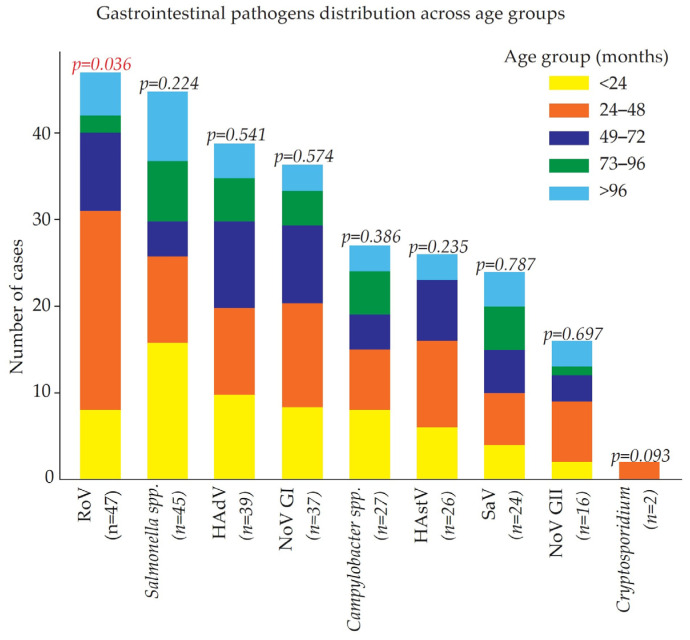
Age distribution of gastrointestinal pathogens among children. The table shows the frequency of cases caused by each pathogen across different age groups and the corresponding *p*-values to indicate statistical significance.

The age distribution of children with AGE caused by different pathogens is presented in Figure 2. Among the various pathogens, RoV demonstrated a statistically significant association with the age group (*p* = 0.036), with the highest prevalence observed in children aged ≤4 years (23 cases), followed by those ≤2 years (eight cases). In contrast, *Salmonella* spp. infections were more evenly distributed across the age groups, with a slight predominance in children ≤2 years (16 cases), but no significant age-related association was detected (*p* = 0.224). HAdV, NoV GI, *Campylobacter* spp., HAstV, and SaV were more commonly detected in children under six years of age, but these associations were not statistically significant (*p* > 0.05). NoV GII was predominantly found in children ≤4 years (seven cases) but did not show a significant correlation with age (*p* = 0.694).

Parasitic infections, such as *Cryptosporidium*, were rare in this cohort, with only two cases reported in children ≤4 years. While a trend toward younger age groups was observed for most viral infections, the overall distribution of bacterial and parasitic infections did not demonstrate significant age-related differences. These findings suggest that RoV remains the most common AGE-associated pathogen in younger children, particularly those under four years of age, whereas bacterial and other viral pathogens exhibit a more even distribution across age groups.

### 3.5. Clinical Symptoms Associated with Specific Pathogens

The clinical manifestations, including diarrhea, fever, vomiting, abdominal pain, and nausea, were analyzed for each pathogen (Table 3). All patients (100%) presented with diarrhea, regardless of the causative pathogen. Fever was a common symptom across infections, ranging from 37.5% in NoV GII cases to 100% in *Cryptosporidium* infections. Among bacterial infections, *Salmonella* spp. had the highest incidence of fever (73.3%), followed by *Campylobacter* spp. (70.4%). Among viral infections, RoV (70.2%) and HAdV (69.2%) were the most frequently associated with fever. However, no statistically significant differences in fever occurrence were observed among the pathogens (*p* = 0.233). Vomiting was most frequently reported in HAstV infections (26.9%), followed by HAdV (25.6%) and NoV GI (24.3%).

Among bacterial pathogens, *Salmonella* spp. (8.9%) and *Campylobacter* spp. (14.8%) were associated with lower vomiting rates. *Cryptosporidium* infections were not linked to vomiting. There was no statistically significant association between vomiting and specific pathogens (*p* = 0.787). Abdominal pain was less commonly reported across cases, with the highest incidence observed in SaV (20.8%), *Campylobacter* spp. (18.5%), and *Salmonella* spp. (15.6%) infections. The lowest occurrence was seen in RoV cases (4.3%). No statistically significant differences in abdominal pain were found among different pathogens (*p* = 0.514). Nausea was observed in 18.8% of NoV GII cases, followed by 17.8% of *Salmonella* spp. infections. SaV (16.7%) and *Campylobacter* spp. (11.1%) also had a notable prevalence of nausea. In contrast, HAstV (11.5%), HAdV (10.3%), and RoV (10.6%) exhibited lower rates of nausea. The association between nausea and specific pathogens approached statistical significance (*p* = 0.053). Overall, while some variations in clinical symptoms were observed among the different pathogens, no statistically significant differences were detected across fever, vomiting, or abdominal pain. However, nausea showed a borderline association with certain pathogens.

## 4. Discussion

Our study provides an updated epidemiological overview of pediatric AGE in Jordan, emphasizing the distribution of pathogens by age group and clinical presentation. The findings indicate that NoV, RoV, *Salmonella* spp., and HAdV are the predominant etiological agents, with variations in prevalence based on the patient’s age and symptom severity. These results align with data from neighboring Middle Eastern countries such as Saudi Arabia and Lebanon, where rotavirus and norovirus remain the predominant causes of pediatric AGE [27,28], as well as global surveillance data reported by the WHO and GEMS [29,30], reinforcing the regional burden of these pathogens in childhood gastroenteritis.

Our study contributes to the growing body of literature on enteric infections in Jordan by identifying key viral and bacterial pathogens associated with AGE. Consistent with previous studies, RoV remains a leading cause of diarrhea in Jordanian children. The 1993 study at Princess Basma Teaching Hospital (which also covered the area of Northern Jordan) reported RoV in 18.9% of cases by electron microscopy and 39.6% by ELISA, while a later study (year 2000) at PRH (a pediatric hospital also covering the area of Northern Jordan) detected RoV in 32.5% of cases using both traditional and molecular techniques [31,32]. Jordan introduced rotavirus vaccination in 2015, and the national coverage had reached approximately 96.4% by 2023, according to the Ministry of Health and UNICEF reports [26]. Despite high coverage, breakthrough infections and waning immunity may account for the continued RoV detection in our cohort.

The 2022 study demonstrated a broader spectrum of enteric viruses, with NoV, HAstV, HAdV, and bocavirus identified alongside RoV [33]. In contrast, our data may reveal a different distribution of viral pathogens, potentially due to variations in diagnostic methods or seasonal influences. Earlier studies in Jordan, such as Nimri et al. (2004) and Khuri-Bulos et al. (2006), primarily relied on antigen detection or culture, which have lower sensitivity compared to the PCR-based methods used in our study [34,35]. This likely contributed to the underreporting of viral agents such as norovirus.

Bacterial pathogens are also significant contributors to diarrhea in Jordan. The study by Youssef et al. (2000) identified enteropathogenic *E. coli* (12.8%), enteroaggregative *E. coli* (10.2%), and *Shigella* spp. (4.9%) as common enteric pathogens, findings that align with those of Meqdam et al. (2022) and the Bedouin population study by Nimri and Meqdam (2004) [32,33,34]. These studies also highlighted *Shigella* spp., *Campylobacter jejuni*, and *Yersinia enterocolitica* as key bacterial causes of diarrhea [32,33,34]. Our study builds on this knowledge by further examining the bacterial contribution to acute gastroenteritis (AGE) and underscoring the value of integrating both traditional culture and molecular diagnostics for enhanced pathogen detection.

The Bedouin study highlighted the significance of parasitic infections, particularly *Giardia lamblia*, *Cryptosporidium* spp., and *Entamoeba histolytica*, which were detected in 50% of patients [34]. These findings emphasize the role of socioeconomic and environmental factors, such as water quality and hygiene, in diarrheal disease transmission. Our study may contribute to this understanding by further exploring the prevalence of parasitic infections and their clinical impact in different populations within Jordan.

In our cohort, RoV was most frequently detected in children aged 2–4 years, with peak incidence at 36 months, mirroring previous local and global trends [31,32,33,36]. Studies from Saudi Arabia, Egypt, and Turkey reported similar age distributions, with the RoV infection rates being the highest in infants and toddlers, while declining in older children [37,38,39]. In Saudi Arabia, RoV cases are most common in children under three years, comprising 60–70% of detected infections, while, in Egypt and Qatar, peak incidence occurs between 6 and 18 months [27,40,41]. This pattern is also observed in Europe and the United States, where RoV infections primarily affect children under five, particularly before routine vaccination [42,43]. Despite the introduction of the RoV vaccine in Jordan in 2015, our study found that RoV remains a leading cause of AGE in young children, suggesting potential gaps in vaccine coverage or effectiveness.

NoV infections exhibited a relatively uniform age distribution, affecting both infants and older children, with peak incidence under 4 years. This trend aligns with studies from the MENA region, where NoV is detected across a wide pediatric age range, often in both community and hospital settings [41,44,45]. Globally, NoV is a leading cause of AGE in children under five, with outbreaks frequently reported in daycare centers and schools [46,47]. Our findings reinforce its role as a significant contributor to gastroenteritis in Jordan, warranting further surveillance and preventive strategies.

*Salmonella* spp. were most prevalent in children under 4 years, with a notable increase in cases beyond infancy. These findings do not align with a previous study conducted in Northern Jordan, which reported a prevalence of 4.5% and peak occurrence in children aged one year [32]. This trend is consistent with findings from Iran and Turkey, where *Salmonella* infections are more common in toddlers and preschool-aged children due to increased exposure to contaminated food, water, and environmental sources [48,49]. In contrast, European and North American studies report a broader age distribution of *Salmonella* infections, often linked to outbreaks associated with specific food sources [50]. The high prevalence of *Salmonella* in our study may reflect local transmission dynamics, including foodborne exposure and hygiene practices.

HAdV infection was also uniformly distributed across all age groups. This is consistent with studies from Turkey and Saudi Arabia, where HAdV-associated AGE predominantly affects children under five [27,39]. HAdV infections are also reported in up to 20% of pediatric AGE cases in Egypt and Saudi Arabia, with similar age-related patterns [37,38]. Unlike RoV and NoV, which are primarily seasonal, HAdV infections have been reported year-round in previous studies [37,39]. However, our study included samples collected only from November to February over a two-year period, limiting our ability to assess seasonal variability. As a result, we cannot draw conclusions regarding seasonal trends based on the data generated in this work.

Symptom severity varied across pathogens, with RoV infections associated with the most severe clinical presentations, including prolonged diarrhea (>5 days), dehydration, and vomiting. These findings align with studies from several countries in the MENA region, where RoV-related hospitalizations are more frequently linked to severe dehydration than bacterial or other viral AGE [51]. NoV cases, while also characterized by vomiting and watery diarrhea, were generally less severe but had a longer duration than HAdV-related AGE. *Salmonella* spp. infections were associated with fever, abdominal pain, and mucoid or bloody diarrhea, a symptom profile consistent with reports from Turkey and Iran, where invasive *Salmonella* strains contribute to more severe enteric illness [48,49]. Finally, viral co-infections were common in our study. RoV and HAdV co-infections were frequently observed and associated with prolonged diarrhea, while *Salmonella* spp. and *Campylobacter* spp. co-infections were associated with severe abdominal pain and bloody diarrhea. Similar co-infection patterns have been reported in Qatar and Turkey, where mixed viral and bacterial infections lead to more severe clinical outcomes [41,52].

Overall, our findings highlight the age-related burden of different gastroenteric pathogens in Jordan, emphasizing the need for targeted interventions. The persistence of RoV despite vaccination suggests a need to assess vaccine coverage and effectiveness. Jordan uses the monovalent Rotarix vaccine in a two-dose schedule. Although the national coverage is high, reaching 96.4% in 2023, individual patient-level vaccination data were unavailable in our study, representing a limitation. The continued detection of RoV suggests possible waning immunity, missed doses, or genotype mismatch, warranting further investigation. The high prevalence of *Salmonella* spp. in toddlers and preschool-aged children underscores the importance of food safety and hygiene practices. The widespread detection of NoV and HAdV highlights their role in pediatric AGE and the potential need for broader surveillance and preventive measures. Comparing our results with regional and global data confirms that Jordan shares common epidemiological trends with neighboring countries, reinforcing the need for coordinated public health strategies to mitigate the impact of childhood gastroenteritis.

This study has several limitations. It was conducted at a single center in Northern Jordan with a relatively small sample size, which may limit the generalizability of the findings. Nevertheless, it provides preliminary insights into the etiology of AGE requiring hospitalization in Jordanian children and lays the groundwork for future multicenter studies. Sampling was restricted to the winter season, potentially missing seasonal fluctuations in pathogen prevalence. Differences between the two winter periods may reflect inter-annual variability in circulating pathogens due to climatic or immunity-related factors. Expanded, year-round surveillance is needed to better capture seasonal trends. Additionally, budget constraints limited the subtyping of NoV and RoV, restricting our ability to assess strain-specific differences. Future studies with broader geographic coverage, larger cohorts, and enhanced molecular diagnostics are essential for a comprehensive understanding of diarrheal disease epidemiology in Jordan.

## 5. Conclusions

This study identifies NoV, RoV, and HAdV as the leading causes of AGE among hospitalized children in Northern Jordan, with frequent viral co-infections. Bacterial and parasitic pathogens played a minimal role in the disease burden. These findings highlight the need for continued surveillance and enhanced vaccination efforts, particularly for RoV, to reduce AGE-related hospitalizations in children.

## Figures and Tables

**Table 1:** Demographic and clinical characteristics of study participants.

Variable	N = 195 (%)
Gender	
Male	116 (59.5)
Female	79 (40.5)
Age (months)	
≤24	46 (23.6)
25–48	60 (30.8)
49–72	30 (15.4)
73–96	30 (15.4)
>96	29 (14.8)
Symptoms during sample collection	
Diarrhea	195 (100)
Fever	130 (66.7)
Vomiting	124 (63.6)
Abdominal pain	97 (49.7)
Nausea	71 (36.4)

**Table 2:** Co-infections among gastrointestinal pathogens. The table presents the number of cases where co-infections occurred between different pathogens. Rows and columns represent pathogens, with intersections indicating the frequency of co-infections.

	RoV	*Salmonella* spp.	HAdV	NoV GI	*Campylobacter* spp.	HAstV	SaV	NoV GII	*Cryptosporidium*
RoV (n = 47)		0	13	12	0	10	3	2	0
*Salmonella* spp. (n = 45)	0		0	0	20	0	0	0	2
HAdV (n = 39)	13	0		7	0	6	1	5	0
NoV GI (n = 37)	12	0	7		0	17	9	4	0
*Campylobacter* spp. (n = 27)	0	20	0	0		0	0	0	0
HAstV (n = 26)	10	0	6	17	0		7	3	0
SaV (n = 24)	3	0	1	9	0	7		1	0
NoV GII (n = 16)	2	0	5	4	0	3	1		0
*Cryptosporidium* (n = 2)	0	2	0	0	0	0	0	0	

**Table 3:** Clinical symptoms associated with gastrointestinal pathogens. The table presents the frequency and percentage of symptoms observed in cases caused by each pathogen. Percentages are calculated based on the total number of cases for each pathogen.

Pathogen	Diarrhea N (%)	Fever N (%)	Vomiting N (%)	Abdominal Pain N (%)	Nausea N (%)
RoV (n = 47)	47 (100)	33 (70.2)	9 (19.1)	2 (4.3)	5 (10.6)
*Salmonella* spp. (n = 45)	45 (100)	33 (73.3)	4 (8.9)	7 (15.6)	8 (17.8)
HAdV (n = 39)	39 (100)	27 (69.2)	10 (25.6)	5 (12.8)	4 (10.3)
NoV GI (n = 37)	37 (100)	21 (56.8)	9 (24.3)	4 (10.8)	3 (8.1)
*Campylobacter* spp. (n = 27)	27 (100)	19 (70.4)	4 (14.8)	5 (18.5)	3 (11.1)
HAstV (n = 26)	26 (100)	16 (61.5)	7 (26.9)	0	3 (11.5)
SaV (n = 24)	24 (100)	16 (66.7)	5 (20.8)	5 (20.8)	4 (16.7)
NoV GII (n = 16)	16 (100)	6 (37.5)	3 (18.8)	1 (6.3)	3 (18.8)
*Cryptosporidium* (n = 2)	2 (100)	2 (100)	0	0	0
*p*-value	1.000	0.233	0.787	0.514	0.053

## Data Availability

The data presented in this study are available from the corresponding authors upon request.

## Supplementary Materials

The following supporting information can be downloaded at: https://www.mdpi.com/article/10.3390/v17050657/s1, Figure S1. Timeline of sample collection across the study period. Table S1. Real-time PCR Ct values of positive samples.
