# Supplementary figures and images for: Prevalence of Acute Gastroenteritis Enteropathogens Among Hospitalized Children in Jordan: A Single-Center Study
Source: Viruses. 2025 Apr 30;17(5):657. doi: 10.3390/v17050657 (PMC12116106; doi:10.3390/v17050657)

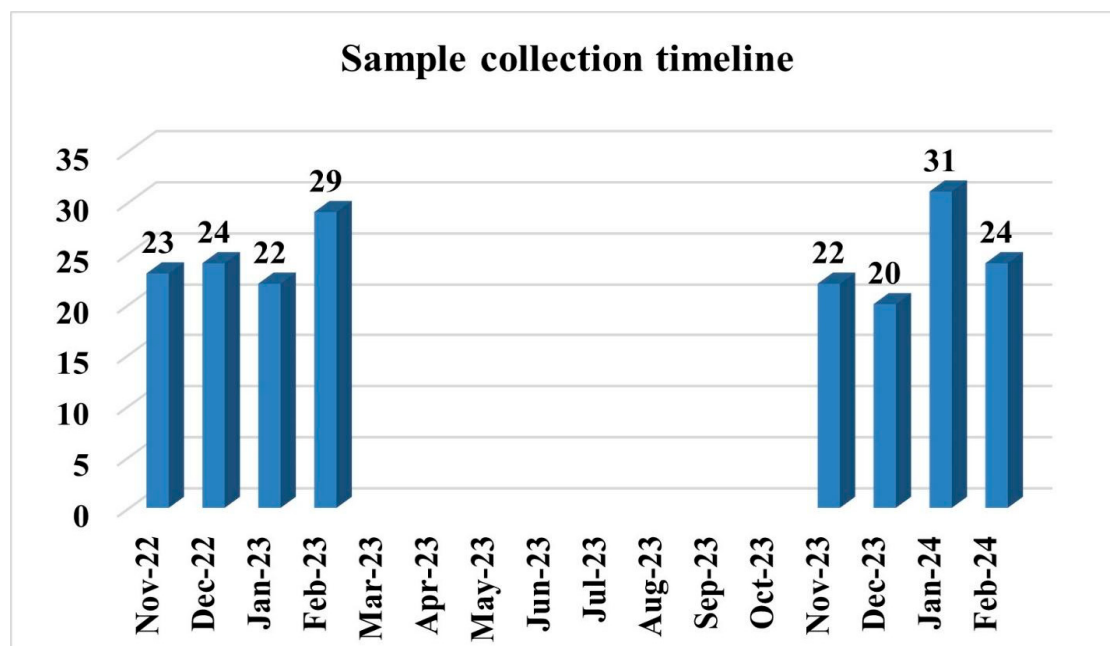

Supplementary Figure S1. Timeline of sample collection across the study period.

Supplement: Supplementary file 1 [file viruses-17-00657-s001.zip › viruses-3580420 - supplementary fig.pdf]
